# GAD1 Gene Expression in Blood of Patients with First-Episode Psychosis

**DOI:** 10.1371/journal.pone.0170805

**Published:** 2017-01-25

**Authors:** Jie Yin Yee, Milawaty Nurjono, Stephanie Ruth Teo, Tih-Shih Lee, Jimmy Lee

**Affiliations:** 1 Research Division, Institute of Mental Health, Singapore, Singapore; 2 Saw Swee Hock School of Public Health, National University of Singapore, Singapore, Singapore; 3 Neuroscience & Behavioural Disorders, Duke-NUS Medical School, Singapore, Singapore; 4 Department of General Psychiatry 1, Institute of Mental Health, Singapore, Singapore; University of Texas Health Science Center at San Antonio Cancer Therapy and Research Center at Houston, UNITED STATES

## Abstract

γ-Aminobutyric acid (GABA), the primary inhibitory neurotransmitter, has often been studied in relation to its role in the pathophysiology of schizophrenia. GABA is synthesized from glutamate by glutamic acid decarboxylase (GAD), derived from two genes, GAD1 and GAD2. GAD1 is expressed as both GAD67 and GAD25 mRNA transcripts with the former reported to have a lower expression level in schizophrenia compared to healthy controls and latter was reported to be predominantly expressed fetally, suggesting a role in developmental process. In this study, GAD67 and GAD25 mRNA levels were measured by quantitative PCR (qPCR) in peripheral blood of subjects with first-episode psychosis (FEP) and from healthy controls. We observed low GAD25 and GAD67 gene expression levels in human peripheral blood. There was no difference in GAD25 and GAD67 gene expression level, and GAD25/GAD67 ratio between patients with FEP and healthy controls. PANSS negative symptoms were associated with levels of GAD25 mRNA transcripts in patients with FEP. While the current study provides information on GAD25 and GAD67 mRNA transcript levels in whole blood of FEP patients, further correlation and validation work between brain regions, cerebrospinal fluid and peripheral blood expression profiling are required to provide a better understanding of GAD25 and GAD67.

## Introduction

Alterations in GABAergic inhibitory neurotransmission have been reported in schizophrenia [1]. *GAD1* is the gene which encodes for a 67kDa isoform of glutamic acid decarboxylase, a key enzyme involved in GABA synthesis, and has been intensively studied [1]. GAD67 was found to be localized in several brain regions, in particular, the prefrontal cortex and hippocampus; its expression gradually increased from 14 weeks of gestational age until approximately 10 years of age and plateauing throughout the rest of lifespan [2,3]. Changes in molecular and cellular mechanisms affecting GAD67 were found to interfere with neuronal activity, connectivity formation during development, glutamatergic and dopaminergic neurotransmission and neurotrophin or glycoprotein signaling [1]. Studies comparing GABA levels in subjects in schizophrenia suggested that a GABA synthesis deficit maybe the cause of cognitive dysfunction in schizophrenia and not unlikely the consequence of the disorder itself [4]. This hypothesis was supported by reports of reduction in GAD67 expression levels in brain regions obtained from postmortem collections in patients with schizophrenia [1].

GAD25, another *GAD1* transcript, lacks an enzymatic domain and is predominantly expressed in human fetal prefrontal cortex and hippocampus, as well as in the olfactory bulb in adult mouse model [3,5]. Its localization suggests possible roles in cell proliferation, migration, maturation of neuroblasts, synaptogenesis and synaptic plasticity [3,5]. As both GAD25 and GAD67 leads to the synthesis of GABA, therefore GAD25:GAD67 ratio may be an indicator of the maturation state of GABA function in human prefrontal cortex and hippocampus [3]. In normal individuals, Hyde et al reported a period of rapid decline in GAD25:GAD67 ratio beginning from fetal development to the first decade of life before it remains stable across the lifespan [3]. However, GAD25:GAD67 ratio was increased in the hippocampus of adult schizophrenics, reflecting an immature state of GABA system [3].

There has been a continuous effort to identify suitable biomarkers for the diagnosis, prognosis and personalization of treatments in people with psychosis [6,7]. However, obtaining brain samples in living individuals for research is a challenge, and it has been done under opportunistic circumstances. However, serial sampling of human brain tissues in living individuals to examine the development of the GABA system is probably near impossible and there had been a continuous effort to explore the possibility of using blood as a peripheral surrogate of brain regions [8]. The present study is the first attempt to explore levels of GAD25 and GAD67 mRNA transcripts and their relative expression in human peripheral blood. We hypothesized that FEP patients would have a higher GAD25/GAD67 ratio than matched controls, suggesting a potentially immature GABA system. We also hypothesized that there will be an association between GAD25 and GAD67 gene expression levels and symptom severity in the FEP patient group.

## Materials and Methods

### Study Participants

The current case control study was conducted at the institute of Mental Health, Singapore. Cases were a mixture of males and females diagnosed with FEP who have had less than 4 weeks of antipsychotic treatment. Controls were healthy individuals with no history of mental illness. Controls were matched for age, ethnicity and gender. Socio-demographic data such as age, gender, ethnicity and smoking status were obtained from all study participants. Participants with a history of substance use, mental retardation and neurological disorders were excluded from this study. Ethics approval for the study was provided by the Domain Specific Review Board of the National Healthcare Group, Singapore. Written informed consent was obtained from all study participants.

### Assessments

All FEP participants were assessed using the Structured Clinical Interview for DSM-IV-TR (SCID-I). Clinical symptoms were assessed on the Positive and Negative Syndrome Scale (PANSS) by trained raters with established inter-rater reliability at > 0.8. Controls were assessed on the SCID-I to determine history of mental illness at recruitment.

### Quantitative Polymerase Chain Reaction

#### RNA isolation

3ml of venous blood was collected from all study participants into Tempus^TM^ Blood RNA tube (Applied Biosystems, Foster City, CA) and stored in -80°C until RNA extraction. Total RNA was extracted using Tempus^TM^ Spin RNA Isolation Kit (Applied Biosystems, Foster City, CA) according to the manufacturer’s protocol. The concentration of extracted total RNA and purity ratio (260/280 and 260/230) were measured using NanoDrop ND-1000 spectrophotometer (Fisher Scientific, Oslo, Norway). The 260/280 ratio of all samples were > 2.1. The quality of total RNA was assessed on ethidium bromide (EtBr)-stained agarose gel. Only samples with clearly defined ribosomal peaks were used in the study. Total RNA was stored at –80°C for later use.

1 μg of RNA obtained from the RNA extraction procedure was reverse transcribed using the iScript cDNA Synthesis Kit (Bio-Rad Laboratories Inc., Hercules, CA, USA) according to the manufacturer’s protocol on an iCycler analyzer (Bio-Rad Laboratories Inc., Hercules, CA, USA).

#### qPCR

Fast real-time PCR was carried out using fluorescent labeled TaqMan® gene expression assays. Three FAM labeled probes were used: (1) β-Actin as endogenous control (ABI assay ID: HS99999903_m1), (2) GAD25 as target (ABI assay ID: HS00247564_m1), (3) GAD67 as target (ABI assay ID HS01065886_m1). The assays were conducted on an ABI7900HT analyzer (Applied Biosystems, Foster City, CA) according to the manufacturer’s protocol. cDNA sample was added to a master mix containing 2X TaqMan® Fast Advanced Master Mix, 20X TaqMan® Gene Expression Assay and DEPC water. Relative quantification using the ΔΔCt method was selected as the assay type. All three genes were run in parallel and in triplicates on the same 96-well plate for each sample. The PCR amplification was carried out under the following conditions: 2 minutes at 50°C (UNG incubation), 20 seconds at 95°C (polymerase activation), and 40 cycles with 1 second at 95°C and 20 seconds at 60°C. Results were analyzed on the SDS and RQ Manager Version 1.2 software (Applied Biosystems, Foster City, CA) which reported the comparative CT values according to the 2^ΔΔ-Ct^ formula.

### Statistical Analysis

Data was analyzed on SPSS Statistics version 23 (IBM Co., Armonk, NY, USA). Descriptive statistics were tabulated for case and control groups. Statistical significance was set at p<0.05. Categorical variables were examined using chi-squared test. Continuous variables were analyzed via independent Student’s t-test or Mann-Whitney U test and data were reported as mean and standard deviation (SD). Univariate linear regression was used to identify variables [age, gender, smoking status and total daily chlorpromazine (CPZ) equivalent] associated with the expression of GAD25 and GAD67. Predictors with p-values 0.2 or less will be included in the final multivariate linear regression model for examining the association of GAD25 and GAD67 expression levels with symptom severity using a PANSS 5-factor model that was previously validated in our local datasets [9]. Only Ct values less than 40 were used for comparison between controls and patients, and linear regression analysis as Ct values above 40 would suggest no amplification according to manufacturer’s protocol.

## Results

57 pairs of FEP patients and healthy controls, matched for age, gender and ethnicity, were recruited for current study. As shown in Table 1, there was no significant difference in age, gender, ethnicity and smoking status between cases and controls.

**Table 1:** Study participants’ demographics.

	Patients	Controls	*p*-value
N	57	57	
	n (%)	n (%)	
Gender			0.851
Male	30 (52.6%)	29 (50.9%)	
Female	27 (47.4%)	28 (49.1%)	
Ethnicity			1.000
Chinese	36 (63.2%)	36 (63.2%)	
Malay	17 (29.8%)	17 (29.8%)	
Indian	4 (7.0%)	4 (7.0%)	
Smoking	16 (28.1%)	13 (22.8%)	0.519
Age, years	29.26 (7.94)	28.96 (7.91)	0.841
Duration of illness, years	1.80 (4.01)		
PANSS Total	64.63 (15.11)		

To explore the feasibility of using blood as a suitable surrogate for *GAD1* gene expression in the central nervous system, qPCR was employed to assess the gene expression profiles of GAD25 and GAD67 in blood of healthy controls and patients with FEP. The lowest Ct values of GAD25 for controls and FEP patients were 31.04 and 31.71, respectively. The lowest Ct values for GAD67 for controls and FEP patients were 33.00 and 33.41, respectively. More than 50% of both patients and controls had Ct values above 35 for both *GAD1* transcripts, suggesting low expression levels in human peripheral blood. On the other hand, β-actin, the reference gene, showed low Ct values, indicating sufficient cDNA template for qPCR assay.

**Fig 1:**
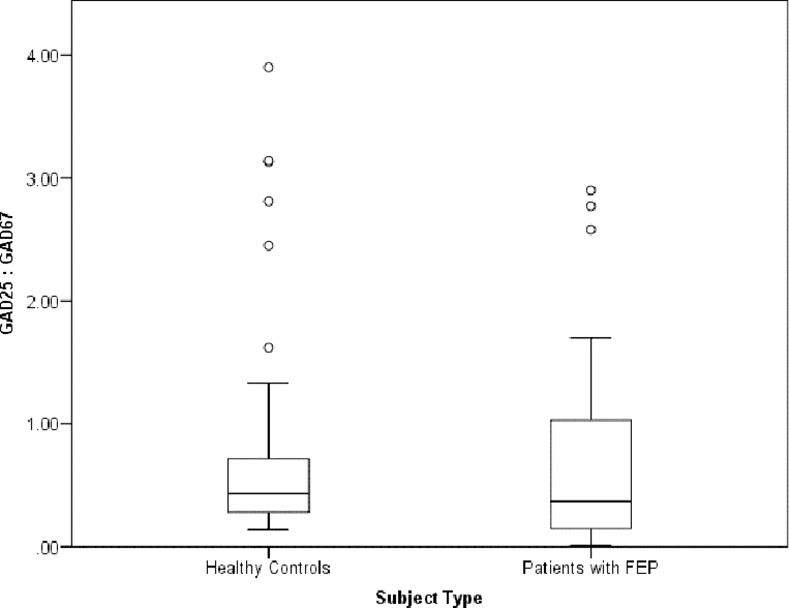
Boxplot of GAD25/GAD67 gene expression levels of patients and controls.

We noted a slight increase in GAD25 (Mann-Whitney U = 968, p = 0.722) and GAD67 (Mann-Whitney U = 640, p = 0.783) fold changes, and GAD25/GAD67 ratio (Mann-Whitney = 460.5, p = 0.212) in patients with FEP but these differences were not statistically significant when compared to healthy controls (Fig 1) (Table 2). Factors known to affect *GAD1* expression levels were determined using univariate linear regression with GAD25 and GAD67 expression levels as dependent variables and age, gender, smoking status and total daily CPZ equivalents as independent variables. Significant predictors for GAD25 were age (F = 3.196, df = 1,88, p = 0.077, R^2^ = 0.035) and smoking (F = 2.297, df = 1,88, p = 0.133, R^2^ = 0.025) (Table 3). Significant predictors for GAD67 were age (F = 3.863, df = 1,70, p = 0.053, R^2^ = 0.052), gender (F = 2.896, df = 1,70, p = 0.093, R^2^ = 0.040) and CPZ equivalents (F = 7.513, df = 1,35, p = 0.010, R^2^ = 0.177) (Table 3).

**Table 2:** Expression levels of GAD25 and GAD67 mRNA.

	Patients	Controls	*p*-value
GAD25 Fold Change			
Mean (SD)	9.49 (12.13)	7.83 (7.21)	0.722
< Ct35	26 (46.4%)	26 (48.1%)	
Ct35—Ct39	18 (31.2%)	20 (37.0%)	
≥ Ct40	12 (21.4%)	8 (14.8%)	
GAD67 Fold Change			
Mean (SD)	25.43 (34.35)	24.63 (31.29)	0.783
< Ct35	21 (37.5%)	13 (24.5%)	
Ct35—Ct39	17 (30.4%)	21 (39.6%)	
≥ Ct40	18 (32.1%)	18 (35.8%)	
GAD25:GAD67	0.70 (0.78)	0.88 (1.03)	0.212

**Table 3:** The association between GAD25 and GAD67 gene expression levels and its predictors.

	Age		Gender		BMI		Smoking		CPZ Equivalent	
	β	P-value	β	P-value	β	0050	β	P-value	β	P-value
GAD25	0.187	0.077	0.114	0.284	0.089	0.405	-0.159	0.133	0.126	0.414
GAD67	0.229	0.053	0.199	0.093	-0.001	0.995	-0.014	0.905	0.420	0.01
GAD25/67	-0.123	0.325	-0.093	0.458	0.047	0.709	-0.087	0.468	-0.381	0.026

Associations between expression levels of GAD25 and GAD67 with symptom severity were examined via multivariate linear regression. Significant predictors mentioned above were included in the models (Tables 4 and 5). After adjusting for the predictors of GAD25 and GAD67 expression levels, there was a significant association between GAD25 expression level and negative symptom factor score (F = 2.790, df = 5,38, p = 0.002, R^2^ = 0.269) (Table 4).

**Table 4:** Association between GAD25 and psychopathology.

GAD25	Unadjusted			Adjusted for age, gender, smoking and CPZ equivalent		
	β	P-value	95% CI	β	P-value	95% CI
Positive	0.035	0.821	-0.583 to 0.731	0.088	0.633	-0.570 to 0.942
Negative	0.437	0.003	0.476 to 2.179	0.479	0.002	0.555 to 2.358
Excitement	-0.155	0.315	-2.379 to 0.786	-0.134	0.421	-2400 to 1.024
Depression	-0.175	0.256	-1.692 to 0.462	-0.102	0.561	-1.594 to 0.878
Cognitive	0.264	0.084	-0.199 to 3.043	0.270	0.101	-0.298 to 3.211

**Table 5:** Association between GAD67 and psychopathology.

GAD67	Unadjusted			Adjusted for age, gender, smoking and CPZ equivalent		
	β	P-value	95% CI	β	P-value	95% CI
Positive	-0.392	0.016	-4.164 to -0.449	-1.465	0.186	-3.672 to 0.742
Negative	0.266	0.112	-0.567 to 5.202	1.768	0.226	-1.159 to 4.685
Excitement	-0.276	0.098	-8.134 to 0.716	-3.576	0.130	-8.363 to 1.112
Depression	-0.088	0.604	-4.317 to 2.548	-0.923	0.583	-4.312 to 2.467
Cognitive	0.382	0.020	1.003 to 10.895	4.571	0.093	-0.801 to 9.943

## Discussion

While studies in the literature on *GAD1* in psychosis were conducted primarily on postmortem brain samples, the current study is the first to examine *GAD1* expression levels in human blood using qPCR. We found that both transcripts, GAD25 and GAD67, were lowly expressed in human peripheral blood.

There had been varying findings from published reports; while majority observed a reduction in GAD67 expression levels in patients with schizophrenia and mood disorders, two reported that the reduced GAD67 levels were due to subset of GABA neurons and two other studies noted an increase in mRNA level in elderly with schizophrenia [10–17]. It has been reported that progressive switches in expression from GAD25 to GAD67 leads to GABA synthesis and higher GAD25/GAD67 ratios in the hippocampus of individuals with schizophrenia were observed, suggesting an immature GABA physiology [3]. The present study observed no differences in expression levels of GAD25, GAD67 and in GAD25/GAD67 ratio between FEP patients and controls. Some of the possible reasons for the differing findings could be due to different cell or tissue types, subsets of patients analyzed and illness duration. A potential reason might be the different duration of illness and treatment. The mentioned studies had patient groups with chronic schizophrenia with at least 15 years of illness compared to the current study which recruited FEP patients. Given differences in GAD67 mRNA levels noted between subgroups of GABA neurons and low GABA levels in the cerebrospinal fluid in subset of patients diagnosed with schizophrenia, whole blood which is made up of a composite group of cells may exhibit different expression profiles [17–19]. The low gene expression level of *GAD1* in peripheral blood may not accurately reflect the *GAD1* profiles in FEP and healthy controls. A report on the comparability of gene expression in blood and brain suggested GAD1 to be expressed in the prefrontal cortex only [6]. It was then suggested that peripheral blood levels may better correlate with tissues with direct access to blood such as pituitary and hypothalamus [6]. Expression of *GAD1* gene was noted to be highest in the cerebral cortex, hippocampus and cerebellum and lowly expressed in the esophagus, kidney, urinary bladder and testis [19]. The lack of difference in GAD1 gene expression levels between patients and controls may be due to the effects of antipsychotics as patients had underwent 4 weeks of antipsychotic treatment prior to participation in the study. The present study also lacks genetic information of *GAD1* to further examine its expression profile in blood. Three single nucleotide polymorphisms at 5’ regulatory region of *GAD1* have been associated with decreased expression levels, increased risk for childhood-onset schizophrenia and cortical gray volume loss [20]. An increase in GAD25/GAD67 expression ratio was associated with a *GAD1* single nucleotide polymorphism (rs3749034), with variant alleles predicting the immature GABA signaling pattern (HYDE 2011) [3]. Moreover, decreased expression of *GAD1* was reported in mutant mice with interneuron-specific ablation of NMDA glutamate receptor subunits [21]. A reduction in *GAD1* expression at prefrontal cortex of people with schizophrenia was observed to be associated with three DNA methylation sites at *GAD1* [22].

Our study found that higher levels of GAD25 mRNA expression in the blood were associated with greater negative symptom burden. This suggests that negative symptoms such as emotional and social withdrawal, poor rapport, and motor retardation may be related to immature GABA development.

The strengths of our study include a consistent sampling time for all participants to minimize possible diurnal variation [18]. We also focused on FEP patients, with minimal exposure to antipsychotics which may interfere with GAD67 expression levels [23–25]. Whole blood was collected directly into Tempus^TM^ blood collection tubes which stabilize the transcriptome with proprietary agents coated within the tubes [26].

To the authors’ knowledge, this is the first attempt to measure *GAD1* transcript levels in whole blood of FEP patients. We did not find any significant difference between GAD25 and GAD67 levels between patient and control groups, probably due to low expression levels. Future attempts might pursue prospective blood samples collected from FEP patients to examine changes in expression levels in relation to chronicity of illness, treatment exposure and clinical outcomes. Studies may also be carried out on antipsychotic-naïve schizophrenia patients to assess the treatment effects on *GAD1* gene expression profile. This information may provide in-depth understanding for the development of psychosis.
